# Diphlorethohydroxycarmalol Attenuates Palmitate-Induced Hepatic Lipogenesis and Inflammation

**DOI:** 10.3390/md18090475

**Published:** 2020-09-18

**Authors:** Seon-Heui Cha, Yongha Hwang, Soo-Jin Heo, Hee-Sook Jun

**Affiliations:** 1 Department of Marine Bio and Medical Sciences, Hanseo University, Chungcheongnam-do 31962, Korea; 2 Gachon Medical and Convergence Institute, Gachon Gil Medical Center, Incheon 21999, Korea; sicrios912@naver.com; 3 Jeju Marine Research Center, Korea Institute of Ocean Science and Technology (KIOST), Jeju 63349, Korea; sjheo@kiost.ac.kr; 4 Department of Biology, University of Science and Technology (UST), Daejeon 34113, Korea; 5 Lee Gil Ya Cancer and Diabetes Institute, Gachon University, Incheon 21999, Korea; 6 College of Pharmacy, Gachon University, Incheon 21999, Korea

**Keywords:** hepatic steatosis, lipogenesis, seaweed, polyphenol

## Abstract

Non-alcoholic fatty liver disease (NAFLD) is a common cause of chronic liver disease, encompassing a range of conditions caused by lipid deposition within liver cells, and is also associated with obesity and metabolic diseases. Here, we investigated the protective effects of diphlorethohydroxycarmalol (DPHC), which is a polyphenol isolated from an edible seaweed, *Ishige okamurae*, on palmitate-induced lipotoxicity in the liver. DPHC treatment repressed palmitate-induced cytotoxicity, triglyceride content, and lipid accumulation. DPHC prevented palmitate-induced mRNA and protein expression of SREBP (sterol regulatory element-binding protein) 1, C/EBP (CCAAT-enhancer-binding protein) α, ChREBP (carbohydrate-responsive element-binding protein), and FAS (fatty acid synthase). In addition, palmitate treatment reduced the expression levels of phosphorylated AMP-activated protein kinase (AMPK) and sirtuin (SIRT)1 proteins, and DPHC treatment rescued this reduction. Moreover, DPHC protected palmitate-induced liver toxicity and lipogenesis, as well as inflammation, and enhanced AMPK and SIRT1 signaling in zebrafish. These results suggest that DPHC possesses protective effects against palmitate-induced toxicity in the liver by preventing lipogenesis and inflammation. DPHC could be used as a potential therapeutic or preventive agent for fatty liver diseases.

## 1. Introduction

Nonalcoholic fatty liver disease (NAFLD) is one of the most common causes of chronic liver diseases worldwide and is characterized by fat deposition in the hepatocytes of patients without history of excessive alcohol consumption. NAFLD is also associated with metabolic complications, including obesity, type 2 diabetes, hyperlipidemia, hypertension and metabolic syndrome [1,2]. Although simple fatty liver itself may be considered a benign disorder, it can progress to hepatitis, fibrosis, and eventually lead to irreversible end-stage liver diseases such as cirrhosis and liver cancer. To control the onset and progression of fatty liver, it is important to inhibit lipogenesis in hepatocytes. Increasing evidence indicates that a large number of polyphenols naturally present in fruits and vegetables may be potential candidates for the treatment of NAFLD [3,4].

Free fatty acids (FFAs) contribute to the liver triglyceride (TG) pool, and the primary sources of FFAs are serum FFAs from adipose tissue and dietary fatty acids [5]. Serum FFA levels are elevated in obese subjects [6], and a previous study reported that serum FFA levels are also elevated in patients with NAFLD [7]. Palmitate, a saturated FFA, is the most common circulating FFA. It has been reported that saturated fatty acids induce hepatocyte lipoapoptosis, and palmitate is more toxic than other saturated and unsaturated fatty acids [8]. TG synthesis caused by FFA accumulation results in severe hepatic injury and fibrosis [9]. Therefore, FFAs are considered to be one of the most important factors that play a crucial role in the pathogenesis of NAFLD [10]. However, it remains unclear how palmitate contributes to inflammation and fibrosis in the liver, and what molecular mechanisms are involved in the pathogenesis of NAFLD and in the progression to inflammation and fibrosis.

Diphlorethohydroxycarmalol (DPHC) is a polyphenolic compound from the edible seaweed *Ishige okamurae*. Several studies have shown various biological functions of DPHC, including antioxidant activity [11,12,13], anti-adipogenic activity [14], anti-inflammatory activity [15], and cytoprotective effects in vitro [14,16,17,18,19,20] and in animal models [21]. Although these diverse effects of DPHC have been investigated, no studies have reported its effects on hepatic steatosis. In this study, we investigated the possible protective effect of DPHC against palmitate-induced lipogenesis and inflammation in the liver in vitro and in a zebrafish model.

## 2. Results

### 2.1. DPHC Protects against Palmitate-Induced Lipotoxicity in HepG2 Cells

**Figure 1:**
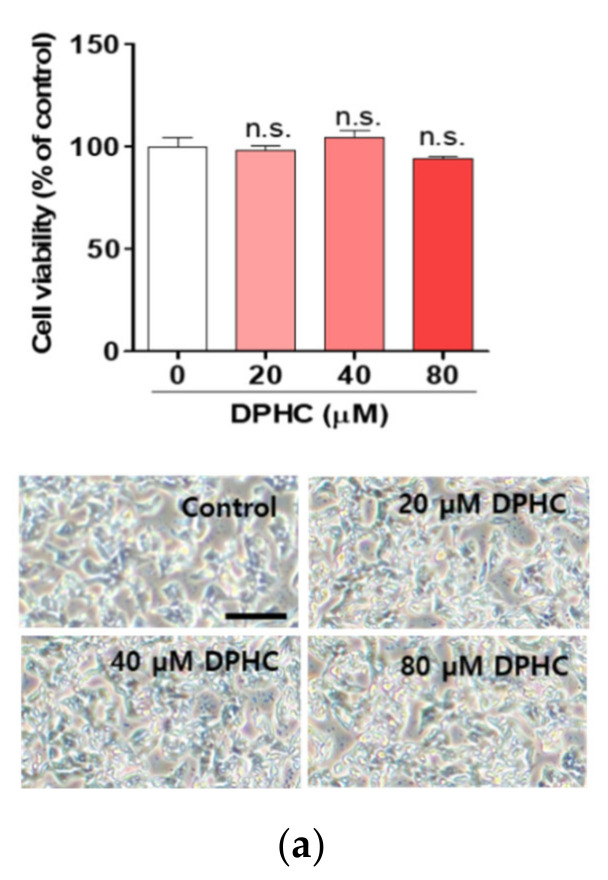

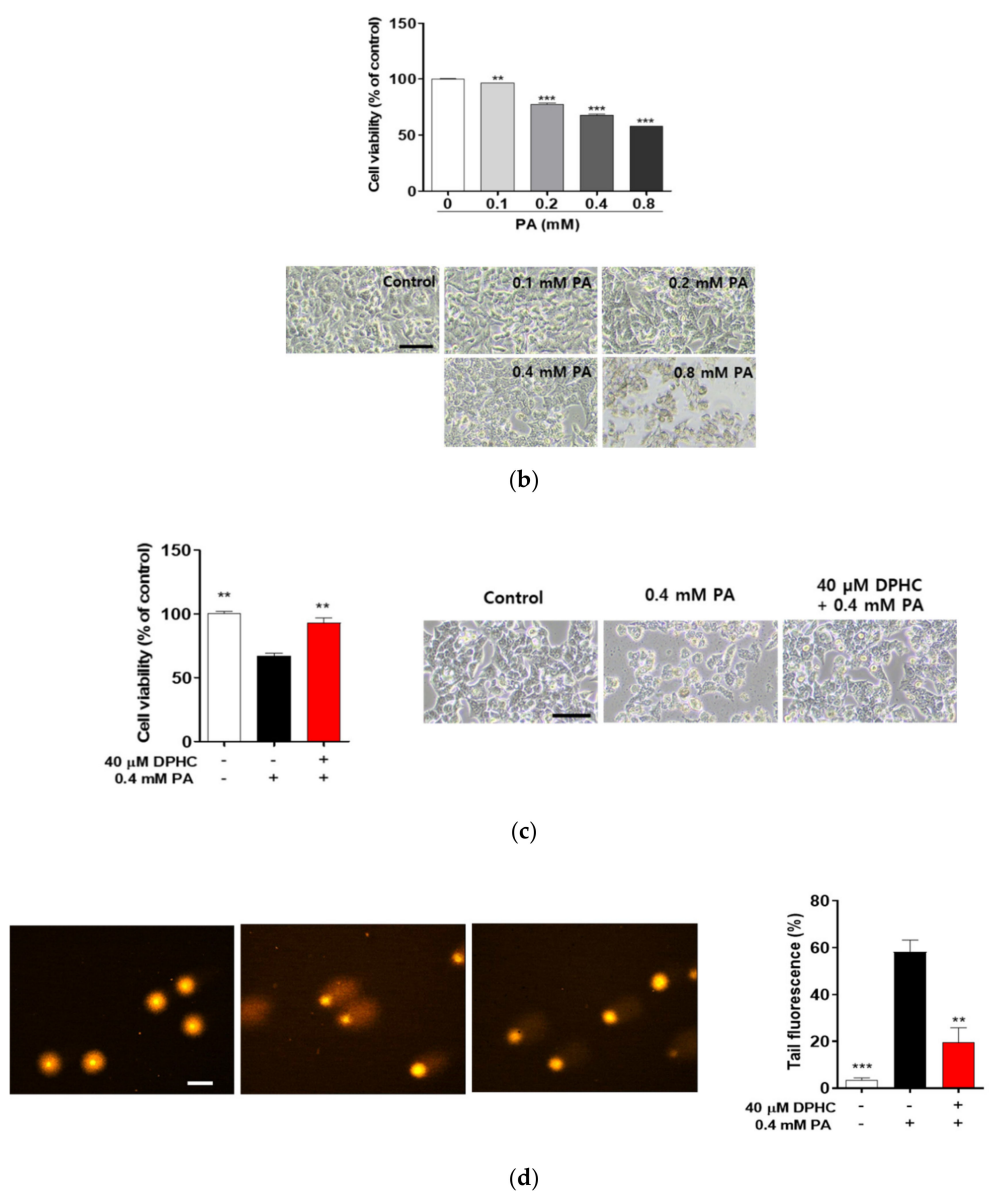
Diphlorethohydroxycarmalol (DPHC) protects against palmitate-induced lipotoxicity in HepG2 cells. (**a**) HepG2 cells were incubated with the indicated concentrations of DPHC for 24 h. (**b**) HepG2 cells were incubated with the indicated concentrations of palmitate (PA) for 24 h. (**c**) HepG2 cells were incubated with and without 40 µM DPHC for 1 h, and then further incubated with or without 0.4 mM palmitate for 24 h. Images were captured at the end of incubation, and CCK-8 assays were subsequently performed. Scale bar indicates 400 µm. (**d**) HepG2 cells were incubated with and without 40 µM DPHC for 1 h, and then further incubated with or without 0.4 mM palmitate for 24 h. DNA damage migration was captured by a fluorescence microscope, and the intensity was measured using Image J. Scale bar indicates 50 µm. Experiments were performed in triplicate. ** *p* < 0.01, *** *p* < 0.001, n.s. indicates no statistical significance.

In order to determine whether DPHC has a protective effect against palmitate-induced toxicity in human hepatocytes, we treated HepG2 cells, a human hepatoma cell line, with palmitate. DPHC alone did not exhibit any toxicity in the cells (Figure 1a). Cytotoxicity was observed in palmitate-treated cells in a dose-dependent manner (Figure 1b). Pretreatment with 40 μM DPHC significantly blocked the cytotoxic effect of palmitate (Figure 1c). To confirm whether DPHC attenuates lipotoxicity, a DNA damage assay was performed, which demonstrated that palmitate induced cellular damage and was protected against by pretreatment of DPHC (Figure 1c), indicating that DPHC possesses a protective effect against palmitate-induced toxicity in HepG2 cells.

### 2.2. DPHC Prevents Palmitate-Induced Lipid Accumulation in HepG2 Cells

**Figure 2:**
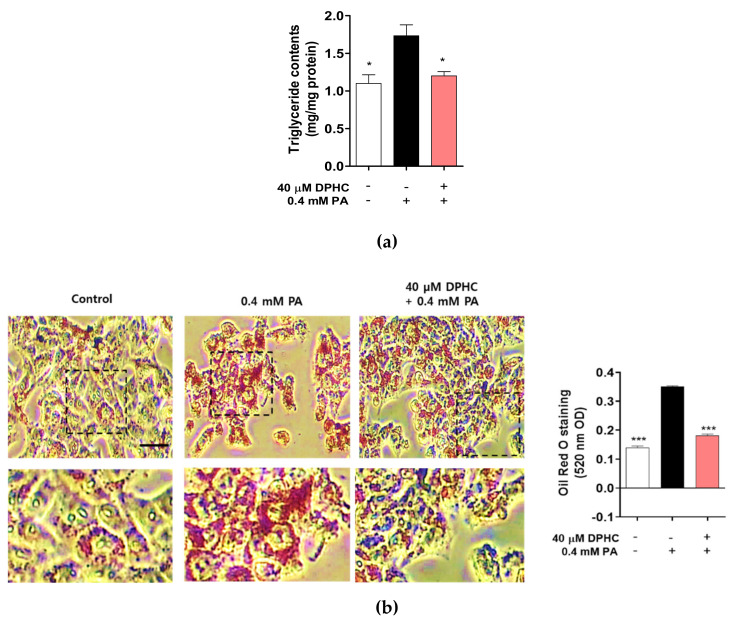
DPHC prevents palmitate-induced lipid accumulation in HepG2 cells. HepG2 cells were incubated with and without 40 µM DPHC for 1 h, and then further incubated with or without 0.4 mM palmitate (PA) for 24 h. (**a**) Triglyceride content. (**b**) Oil Red O staining. Scale bar indicates 50 µm. Experiments were performed in triplicate. * *p* < 0.05, *** *p* < 0.001.

FFAs may play a major role in the development of NAFLD, which is associated with TG accumulation in the liver [22,23]. Therefore, we determined whether DPHC can attenuate the production of palmitate-induced TG accumulation in HepG2 cells. As expected, TG production was significantly increased by palmitate treatment, whereas pretreatment with DPHC significantly reduced palmitate-induced TG accumulation (Figure 2a). In addition, intracellular neutral lipid Oil Red O staining was increased by palmitate treatment, whereas DPHC pretreatment of the cells reduced this lipid droplet accumulation (Figure 2b). These results suggest that DPHC ameliorates palmitate-induced lipogenesis in HepG2 cells.

### 2.3. DPHC Inhibits Palmitate-Induced Lipogenesis-Related Gene Expression in HepG2 Cells

**Figure 3:**
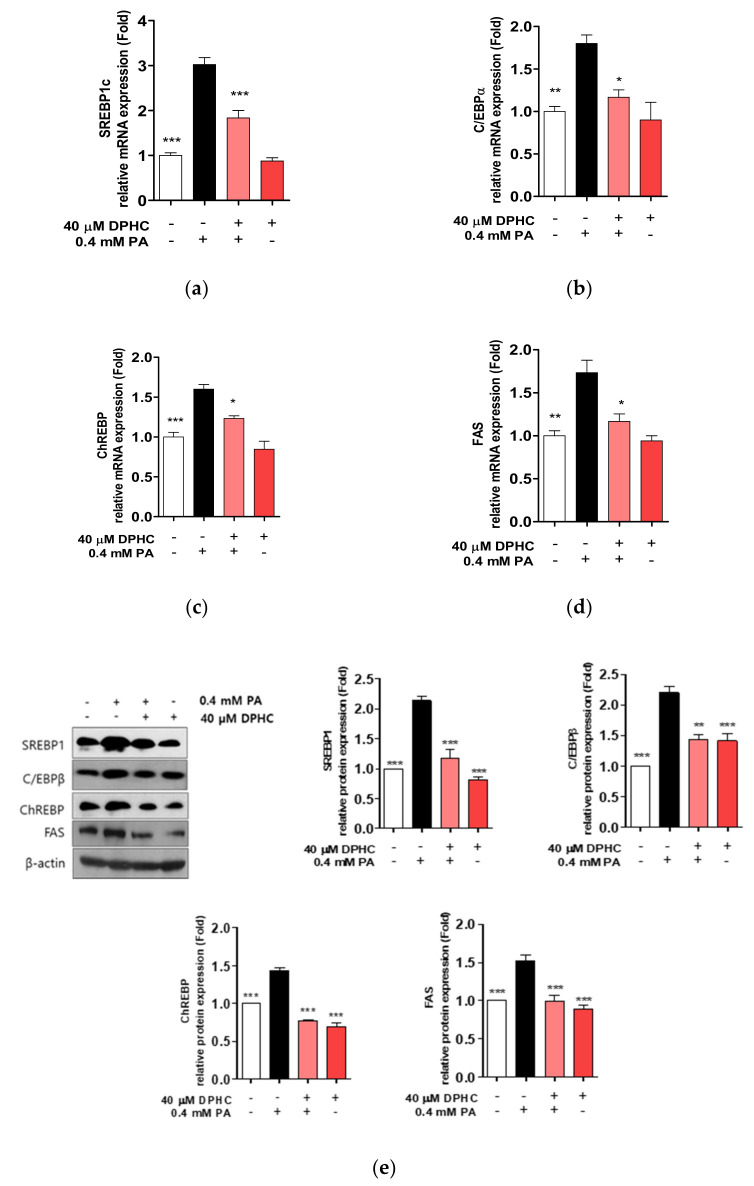
DPHC prevents palmitate-induced lipogenesis in HepG2 cells. Cells were incubated with and without 40 µM DPHC for 1 h, and then further incubated with or without 0.4 mM palmitate (PA) for 24 h. Lipogenesis-related genes (**a**) *SREBP1c*, (**b**) *C*/*EBPβ*, (**c**) *ChREBP*, and (**d**) *FAS* mRNA expression levels were identified by RT-qPCR. (**e**) Lipogenesis-related protein expression detection was performed by western blot. Experiments were performed in triplicate. * *p* < 0.05, ** *p* < 0.01, *** *p* < 0.001.

To examine whether DPHC prevents lipogenesis in HepG2 cells, we examined the mRNA and protein expression of lipogenesis-related genes. We found that the mRNA expression of sterol regulatory element-binding protein (SREBP)1c, CCAAT-enhancer-binding protein (C/EBP)α, carbohydrate-responsive element-binding protein (ChREBP), and fatty acid synthase (FAS) were increased by palmitate exposure, whereas increases in levels of these mRNAs were suppressed, similar to control levels (Figure 3a–d). In addition, protein expression levels of SREBP1, C/EBPβ, ChREBP, and FAS were also increased by palmitate treatment, and this increase was abolished by DPHC pretreatment prior to palmitate treatment (Figure 3e). These results suggest that DPHC may prevent lipid accumulation by inhibiting the expression of lipogenesis-related genes induced by palmitate in HepG2 cells.

### 2.4. DPHC Rescues Palmitate-Induced Reduction of Phosphorylated AMP-Activated Protein Kinase (AMPK) and Sirtuin (SIRT)1 in HepG2 Cells

**Figure 4:**
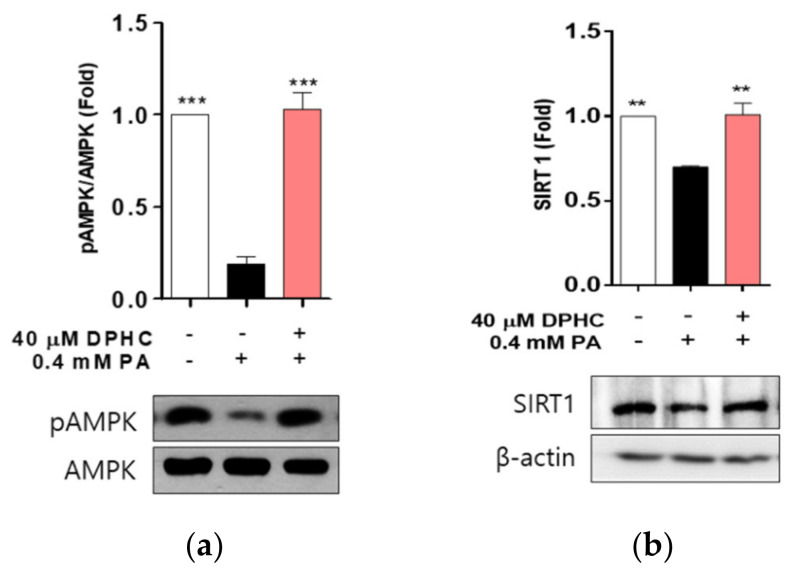
DPHC improves protein expression levels of phosphorylated AMP-activated protein kinase (AMPK) and AMP-activated protein kinase (SIRT)1 against palmitate in HepG2 cells. The cells were incubated with and without 40 µM DPHC for 1 h, and then further incubated with or without 0.4 mM palmitate (PA) for 24 h. (**a**) AMPK and (**b**) SIRT1 protein expression levels were determined by western blotting. Experiments were performed in triplicate. ** *p* < 0.01, *** *p* < 0.001.

AMPK plays a central role in the regulation of lipid metabolism by switching on inhibition of lipid synthesis [24,25], and may be a therapeutic target for treating fatty liver disease. Therefore, we determined whether DPHC stimulates the AMPK signaling pathway. We found that the protein level of phosphorylated AMPK was significantly decreased by palmitate treatment, and this decrease was rescued with DPHC pretreatment of cells (Figure 4a), suggesting that DPHC may upregulate the phosphorylation of AMPK.

The nicotine adenine dinucleotide (NAD(^+^)^−^dependent protein deacetylase activation of SIRT1 is positively associated with the protection of hepatocytes against palmitate-induced lipotoxicity [26], and is currently emerging as a potential therapeutic target for treating fatty liver disease [27,28]. Thus, we determined whether DPHC activates SIRT1, and found that the protein expression level of SIRT1 was decreased by palmitate treatment, and this decrease was rescued by DPHC pretreatment (Figure 4b). These results suggest that DPHC may induce SIRT1 expression, contributing to the prevention of lipotoxicity.

### 2.5. DPHC Protects against Palmitate-Induced Liver Lipogenesis in Zebrafish

**Figure 5:**
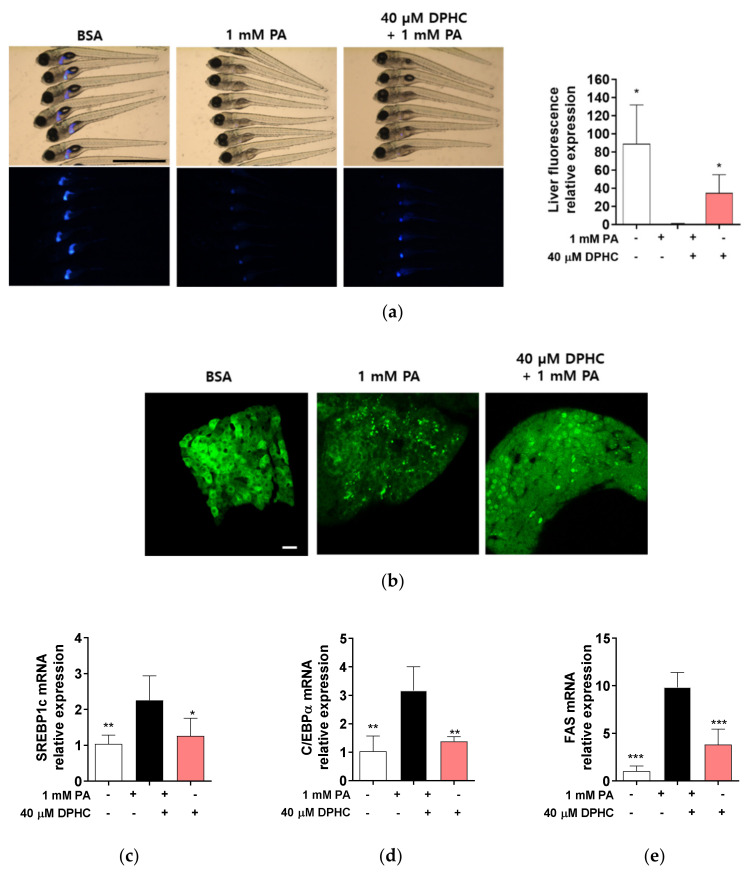
DPHC protects against palmitate-induced liver lipogenesis in zebrafish. At 3 days post-fertilization, zebrafish were incubated with and without 40 µM DPHC for 1 h, and then further incubated with or without 1 mM palmitate (PA) for 72 h. (**a**) Representative phase contrast images of zebrafish and fluorescence microscopy images of the zebrafish liver. Fluorescence relative value was calculated by ImageJ. Scale bar indicates 700 µm. (**b**) Representative confocal microscopy images of the isolated zebrafish liver. Scale bar indicates 10 µm. Total RNA was extracted from zebrafish liver and mRNA expression levels of (**c**) *SREBP1c*, (**d**) C/*EBPβ*, and (**e**) *FAS* were analyzed by RT-qPCR. *n* = 12–15 embryos. * *p* < 0.05, ** *p* < 0.01, *** *p* < 0.001 versus PA-treated group.

To determine whether DPHC directly protects against liver damage in vivo, we used transgenic zebrafish (*Danio rerio*) expressing enhanced green fluorescent protein (EGFP) under the control of the liver fatty acid-binding protein promoter. Zebrafish embryos were preincubated in 40 μM DPHC for 1 h and then further incubated with 1 mM palmitate for 72 h. EGFP expression in the liver was observed to be reduced by palmitate treatment, whereas higher expression of EGFP was observed in DPHC-pretreated embryos (Figure 5a).

Next, we determined whether DPHC can attenuate palmitate-induced lipogenesis-related gene expression in zebrafish embryos. Hepatocytes expression in zebrafish liver was also reduced in palmitate embryos, whereas it was protected against by DPHC pretreatment. As expected, mRNA expression levels of SREBP1c, C/EBP1α, and FAS were significantly increased by palmitate treatment, whereas these increases were reduced by DPHC pretreatment (Figure 5c–e). These results suggest that DPHC may ameliorate palmitate-induced lipogenesis in the liver of zebrafish.

### 2.6. DPHC Protects against Palmitate-Induced Liver Inflammation in Zebrafish

**Figure 6:**
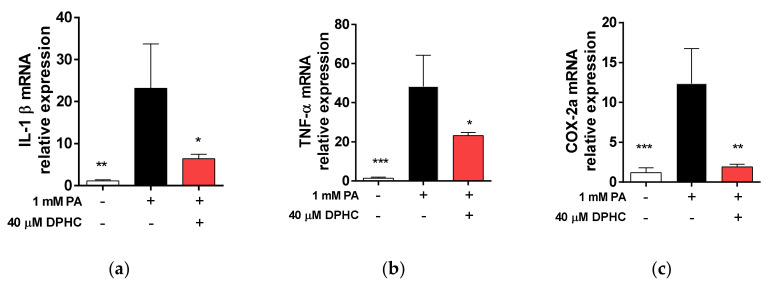
DPHC protects against palmitate-induced liver inflammation in zebrafish. At 3 days post-fertilization, zebrafish were incubated with and without 40 µM DPHC for 1 h, and then further incubated with or without 1 mM palmitate (PA) for 72 h. Total RNA was extracted from zebrafish liver, and mRNA expression levels of (**a**) *IL-1β*, (**b**) *TNF-α*, and (**c**) *COX-2a* were analyzed by RT-qPCR. PA: palmitic acid. *n* = 12–15 embryos. * *p* < 0.05, ** *p* < 0.01, *** *p* < 0.001.

Accumulation of FFAs induces inflammation and causes lipotoxic effects in the liver [6]. Because fatty acid metabolism plays a role in the inflammatory response, we determined whether DPHC attenuates palmitate-induced inflammation in the liver of zebrafish. We identified that mRNA expression levels of interleukin (IL)-1β, tumor necrosis factor (TNF)-α, and cyclooxygenase (COX)-2a were increased by palmitate treatment, whereas these increases were reduced in embryos pretreated with DPHC (Figure 6a–c), suggesting that DPHC may protect against pro-inflammatory cytokine overexpression.

### 2.7. DPHC Protects against Palmitate-Induced Reduction of Phosphorylated AMP-Activated Protein Kinase (AMPK) and Sirtuin (SIRT)1 in Zebrafish

**Figure 7:**
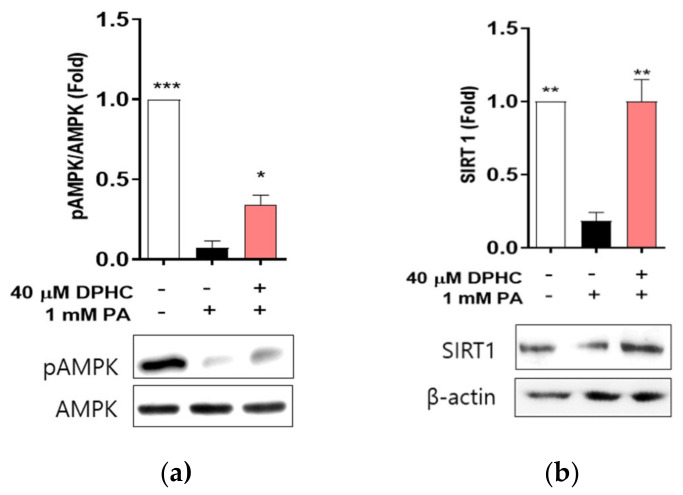
DPHC improves protein expression levels of phosphorylated AMPK and SIRT1 against palmitate in zebrafish liver. Zebrafish embryos were incubated with and without 40 µM DPHC for 1 h, and then further incubated with or without 1 mM palmitate (PA) for 24 h. (**a**) AMPK and (**b**) SIRT1 protein expression levels were determined by western blotting. Experiments were performed in triplicate. * *p* < 0.05, ** *p* < 0.01, *** *p* < 0.001.

We determined whether DPHC stimulates the AMPK and SIRT1 signaling pathway in zebrafish. We found that the protein level of phosphorylated AMPK and SIRT1 was significantly decreased by palmitate treatment, and this decrease was rescued with DPHC pretreatment of zebrafish (Figure 7), suggesting that DPHC may upregulate the phosphorylation of AMPK and SIRT1 in in vivo zebrafish. These results suggest that DPHC may induce AMPK and SIRT1 expression, contributing to the prevention of lipotoxicity in zebrafish liver.

## 3. Discussion

NAFLD is recognized as a global health problem and as a common cause of chronic liver disease. To date, there are two major strategies in NAFLD therapy: (1) lifestyle modification, including dietary modification and physical exercise, and (2) pharmaceutical therapies. Lifestyle modifications with diet and exercise have been recommended as the initial management. However, these changes are difficult to achieve and sustain over time. Therefore, attention has been focused on finding pharmacologic agents for the treatment and/or prevention of NAFLD. Although several drugs to treat NAFLD are currently available, satisfactory outcomes have not been achieved. Therefore, natural products have been considered as alternative treatments to prevent NAFLD, or to stop its progression through several mechanisms, such as the downregulation of pro-inflammatory cytokines, antioxidant effects, or by anti-dyslipidemic properties [29,30,31]. Recently, constituents of seaweed origin have emerged in studies seeking improvements in NAFLD [32,33].

Polyphenolics are a type of flavonoid, which represents the most common group of dietary components, and have been suggested to be consistently associated with a reduced risk of developing chronic diseases, including diabetes mellitus, cardiovascular disease, and inflammation, as well as NAFLD [34,35,36].

Polyphenolic compounds, which are known to exhibit various biological activities, such as antioxidant, anti-inflammatory, and anti-glycation effects, are found in the edible seaweed *Ishige okamurae* [37,38,39]. However, it is not known whether DHPC, one polyphenolic compound from *I. okamurae*, might ameliorate palmitate-induced NAFLD. In the present study, we investigated the effects of DPHC on palmitate-induced lipogenesis, as well as inflammation, in HepG2 cells and zebrafish. Our study demonstrated that palmitate treatment caused hepatic toxicity, increased lipogenesis-related gene expression, and increased levels of pro-inflammatory cytokines. All of these effects were significantly attenuated by pretreatment with DPHC.

FFA is a prominent causative factor of NAFLD [40], and increased FFA levels have been observed in patients with NAFLD [41,42]. FFA induces excessive TG accumulation in hepatocytes, and this alters lipid metabolism in the liver [43]. SREBP-1c is a master transcriptional regulator of lipogenesis, and is highly expressed in the liver [44]. ChREBP is a transcription factor, which is activated by carbohydrate, and induces both glycolysis and lipogenesis [45]. In addition, C/EBPβ is a transcription factor, which is known to be an important regulator in fatty liver disease [46]. We found that palmitate treatment induced fat accumulation and elevated the mRNA and protein levels of SREBP-1c, ChREBP, and C/EBPβ; however, DPHC treatment inhibited these increases in HepG2 cells. Consistent with these results, the expression of *FAS*, a target gene of SREBP-1c and ChREBP, was increased by palmitate treatment, and DPHC pretreatment inhibited this increase. These results indicated that DPHC might have an ameliorating effect on fatty liver through the inhibition of the expression of lipogenic genes.

SIRT1 is a protein belonging to the sirtuin family, which widely affects lipid metabolism with AMPK signaling [47]. SIRT1-AMPK signaling in several metabolic tissues, including the liver, has been reported to increase rates of fatty acid oxidation, and to repress lipogenesis, largely by modulating SREBP-1 [48,49]. Thus, the SIRT1-AMPK axis has emerged as a major signaling system in regulating the lipid-lowering action in tissues, including the liver. The major therapeutic effect of polyphenol supplementation in NAFLD is reported to be mediated by the activation of AMPK [50,51]. The results obtained in this study were comparable, which allowed us to draw the following conclusions: DPHC pretreatment increases AMPK phosphorylation, which entails the downregulation of SREBP-1 expression levels [50]. Furthermore, a number of investigations have revealed that polyphenolic compounds significantly affect lipid metabolism [52], decrease plasma TG [53], and reduce lipid peroxidation [54]. Therefore, we suggest that DPHC, as used in this study, may also regulate lipid metabolism. In addition, the activation of AMPK and SIRT1 by polyphenols [55,56,57] inhibits inflammation [58], as well as suppresses the development of NAFLD [59,60,61]. These reports suggest that polyphenolic compounds have a prominent NAFLD development-alleviating effect.

It is widely acknowledged that hepatocyte inflammatory responses are associated with obesity and involve liver lipid accumulation, subsequently progressing to hepatic steatosis in the course of NAFLD. Lipid accumulation is one of the causative factors for inflammation, and accelerates the progress of NAFLD [62]. Some polyphenols significantly inhibit pro-inflammatory cytokines and transcription factors, including *IL-1, IL-6, TNF-α*, *NF-kB*, and *COX-2,* in the liver [63,64], DPHC is reported to possess anti-inflammatory effects in keratinocytes [15]. Therefore, we determined whether DPHC affects the expression of pro-inflammatory cytokines in the liver of zebrafish. In this study, we observed that DPHC can significantly reduce the expression of pro-inflammatory cytokines in the liver of zebrafish, suggesting that DPHC possesses anti-inflammation functions in NAFLD.

In summary, we demonstrated that DPHC reduced hepatic toxicity, diminished the expression of *SREBP-1, C/EBPβ, ChREBP*, and *FAS*, and enhanced *AMPK* and *SIRT1* expression, as well as reduced hepatic inflammation induced by palmitate. These results suggested that the activation of the AMPK signaling pathway may play a critical role in the suppressive effect of DPHC on lipogenesis, as well as in the promoting effect of DPHC on SIRT1. These findings may provide molecular evidence for the use of DPHC as a therapeutic agent in the management of NAFLD. However, although DPHC showed an excellent hepatic protective effect against PA in the in vivo zebrafish model in this study, it is necessary to confirm whether it is safe in both healthy and in hyperglycemia or obesity state when taking DPHC for a long time as pharmaceutical agent.

## 4. Materials and Methods

### 4.1. Preparation of DPHC from Ishige okamurae

The DPHC isolated from the seaweed *Ishige okamurae* was used. The preparation procedure of DPHC was described in our previous study [40].

### 4.2. Cell Culture

The human liver hepatocellular cell line HepG2 was obtained from the American Type Culture Collection (ATCC, Manassas, VA, USA). The cells were cultured in DMEM (Dulbecco Modified Eagle Medium, Welgene, Kyungsangbuk-do, Korea) supplemented with 10% FBS (Fetal Bovine Serum, Welgene), 100 U/mL penicillin, and 100 μg/mL streptomycin (Welgene), and were maintained in a humidified incubator with 5% CO_2_.

### 4.3. Assessment of Cell Viability

Cell viability was estimated using a cell counting kit (D-Plus™ CCK; Dongin LS, Kyunggi-do, Korea) that measures water-soluble tetrazolium. For the CCK assay, HepG2 cells (2 × 10^4^ cells/well) were seeded into 96-well plates. After 16 h, the cells were treated with DPHC and/or palmitate (Sigma, St. Louis, MO, USA) at 37 °C. D-Plus™ CCK solution was then added to the wells for a total reaction volume of 110 µL. After 2 h of incubation, optical absorbance was measured at a wavelength of 450 nm. The optical density of the formazan generated in control cells was considered to represent 100% viability.

### 4.4. Triglyceride Content Determination

HepG2 cells (2 × 10^5^ cells/well) were seeded into 12-well plates. After 16 h, the cells were treated with DPHC and/or palmitate (Sigma) at 37 °C. TG levels were estimated using a TG quantification kit (BioVision, Milpitas, CA, USA) according to the manufacturer’s protocol. Briefly, cells were dissolved in 5% NP-40–H_2_O, and glycerol converted from TGs was measured.

### 4.5. Oil Red O Staining

The total intracellular lipid content was evaluated by Oil Red O staining. Briefly, cells were fixed in 4% paraformaldehyde in PBS (phosphate buffered saline, Welgene) for 30 min, stained with freshly prepared 0.28% Oil Red O for 30 min at room temperature, and then rinsed with water. Cell images were captured by photomicroscopy (Nikon Eclipse Ts2, Tokyo, Japan) equipped with eXcope XCAM 1080 (DIXI Science, Daejeon, Korea). For quantitative analysis of cellular lipids, 1 mL of isopropanol was added to the stained cells. The extracted dye was removed immediately by gentle pipetting, and its absorbance was read using a spectrophotometer at 510 nm. Data are represented as percentages of control cells.

### 4.6. RT-qPCR

Total RNA was extracted from HepG2 cells and zebrafish liver using RNAiso plus (Takara Bio Inc., Kusatsusi, Japan), and cDNA was prepared using a PrimeScript™ cDNA synthesis kit (Takara Bio Inc.) according to the manufacturer’s instructions. cDNA samples were analyzed using SYBR^®^ Premix Taq™, ROX plus (Takara Bio Inc.) on a Bio-Rad cycler (Hercules, CA, USA). Gene expression was normalized to that of the endogenous housekeeping control gene *β-actin*, which was not influenced by palmitate. Relative expression was calculated for each gene using the ΔΔC_T_ method (where C_T_ is the threshold cycle). The primer sequences used are listed in Table 1.

### 4.7. Western Blotting

HepG2 cells (1 × 10^5^ cells/well) were seeded into six-well plates, and the cells were incubated with vehicle (control) or 40 µM DPHC for 1 h, and then further incubated with or without 0.4 mM palmitate for 24 h. The cells were lysed using 1% Triton X-100-PBS and protease inhibitor cocktail (GenDEPOT, Barker, TX, USA) for 20 min, on ice. The lysates were fractionated by centrifugation at 12,000 rpm for 20 min at 4 °C, and the pellets were used for western blotting. Protein concentrations were measured using a DC protein assay kit (Bio-Rad, Hercules, CA, USA). The lysates were separated by SDS-PAGE and transferred to PVDF (polyvinylidene fluoride) membranes (Millipore, Billerica, MA, USA). Membranes were incubated with 5% skimmed milk for 1 h at room temperature, and then incubated with primary antibodies overnight at 4 °C. After washing extensively, membranes were incubated with horseradish peroxidase-conjugated secondary antibody (Jackson ImmunoResearch, West Grove, PA, USA). Signals were detected using WESTSAVE (Ab Frontier, Seoul, Korea) and an enhanced chemiluminescence system. ImageJ software was used to quantify the band intensities of western blots. The primary antibodies used were anti-SREBP1, anti-C/EBPβ, anti-ChREBP, anti-FAS, and anti-β-actin. All primary antibodies were purchased from Santa Cruz Biotechnology (Santa Cruz, CA, USA).

### 4.8. Zebrafish Experiments

The zebrafish embryo procedures used in the present study were conducted according to the guidelines established by the Gachon University Ethics Review Committee for Animal Experiments.

Transgenic zebrafish embryos expressing EGFP under the control of the liver fatty acid-binding protein promoter *Tg*(*lfabp-egfp*) were obtained from the Korean Zebrafish Organogenesis Mutant Bank. At 3 days post-fertilization (dpf), embryos were arrayed in 12-well plates for experiments. At 3 dpf, embryos (*n* = 12–15) were transferred to 12-well plates and maintained in 1 mL of embryo media (0.003% sea salt, 0.0075% calcium sulfate). Embryos were preincubated with 40 µM DPHC for 1 h, and then further incubated in the presence of 1 mM palmitate for 72 h. Thereafter, the embryos were rinsed in embryo media and anaesthetized using 2-phenoxy ethanol (Sigma) before experiments. The zebrafish were imaged using a stereo fluorescence microscope (M165FC, Leica, Wetzlar, Germany) and confocal microscope (LSM710, Zeiss, Oberkochen, Germany). After isolation of the liver, mRNA expression levels of specific genes were determined.

### 4.9. Statistical Analysis

Significant differences were compared using one-way analysis with subsequent multiple comparison test (Tukey) of variance using GraphPad prism version 6.0 (GraphPad software, San Diego, CA, USA). Data are presented as means ± SEM. Differences were considered significant at *p* < 0.05 versus the PA-treated group.

## Figures and Tables

**Table 1:** Primer sequences.

Gene name	Sequence 5′-3′	
*SREBP1c* (HepG2 cells)	Forward Reverse	TCGCGGAGCCATGGATT GGAAGTCACTGTCTTGGTTGTTGA
C/*EBPα* (HepG2 cells)	Forward Reverse	GACACGCTGCGGGGCATCT CTGCTCCCCTTCCTTCTCTCA
*ChREBP* (HepG2 cells)	Forward Reverse	GTCTGCAGGCTCGGAACAG AAGGAGGAAATCAGAACTCAGGAA
*FAS* (HepG2 cells)	Forward Reverse	GCAAATTCGACCTTTCTCAGAA GTAGGACCCCGTGGAATGTC
Cyclophilin (HepG2 cells)	Forward Reverse	TGCCATCGCCAAGGAGTAG TGCACAGACGGTCACTCAAA
*IL-1β* (Zebrafish)	Forward Reverse	TCAAACCCCAATCCACAGAG TCACTTCACGCTCTTGGATG
*TNF-α* (Zebrafish)	Forward Reverse	AGAAGGAGAGTTGCCTTTACCGCT‘ AACACCCTCCATACACCCGACTTT
*COX-2* (Zebrafish)	Forward Reverse	AGCCCTACTCATCCTTTGAGG TCAACCTTGTCTACGTGACCATA
*FAS* (Zebrafish)	Forward Reverse	GCACCGGTACTAAGGTTGGA CAGACGCCATGTTCAAGAGA
*β-actin* (Zebrafish)	Forward Reverse	AATCTTGCGGTATCCACGAGACCA TCTCCTTCTGCATCCTGTCAGCAA

SREBP1c: sterol regulatory element-binding protein 1; C/EBP: CCAAT-enhancer-binding protein; ChREBP: carbohydrate-responsive element-binding protein; FAS: fatty acid synthase; IL-1: interleukin 1; TNF: tumor necrosis factor; COX: cyclooxygenase.
